# Tumor to Tumor Metastasis: A Case Report

**DOI:** 10.31729/jnma.5699

**Published:** 2021-02-28

**Authors:** Diksha Karki, Purbesh Adhikari, Dipti Shrestha, Anil Kafle

**Affiliations:** 1 Department of Pathology, BP Koirala Institute of Health Sciences, Dharan, Nepal

**Keywords:** *adenocarcinoma*, *case report*, *metastasis*, *uterine leiomyoma*

## Abstract

Colon carcinoma spreads locally around the intestine wall and can undergo distant metastasis via the hematogenous or lymphatic spread. It rarely metastasizes to the female genital tract and is not fully reported to involve a uterine leiomyoma. Herein we report such an unusual case of a 27 years female a known case of sigmoid colon adenocarcinoma who presented with abdominal pain with bilateral adnexal mass and per vaginal bleeding. Exploratory laparotomy with bilateral resection of ovaries with subsequent polypectomy was done, which on histopathology, it revealed metastatic adenocarcinoma in bilateral ovaries and submucosal leiomyoma. Hence, a lesion with dimorphic histomorphology should be carefully evaluated to rule out the possibility of malignant-to-benign tumor-to-tumor metastasis.

## INTRODUCTION

Metastasis to the female genital tract from extragenital malignancy is rare, with ovaries being the commonest site to be affected.^1^ Even rarer is the metastasis within uterine myoma. Fried first reported this unusual phenomenon of one tumor metastasizing to another in 1930.^2^ Any malignant tumor can be a donor tumor, with the most commonly reported being carcinomas of lung followed by breast, gastrointestinal tract, prostate, and thyroid. In contrast, the intracranial meningioma being the commonest reported recipient tumor.^3,4^ Here, we present one of such unusual combinations of recipient and donor tumor, a case of sigmoid colon adenocarcinoma metastasizing to submucosal leiomyoma of the uterus.

## CASE REPORT

**Figure 1.**
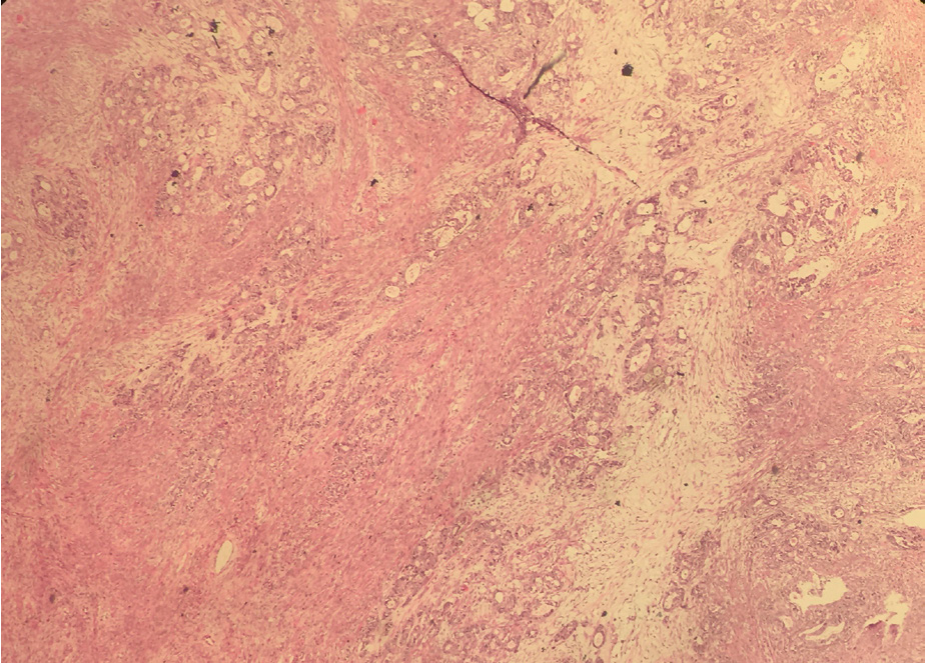
Tumor cells infiltrating the smooth muscle bundle of leiomyoma (H&E, 40x).

**Figure 2.**
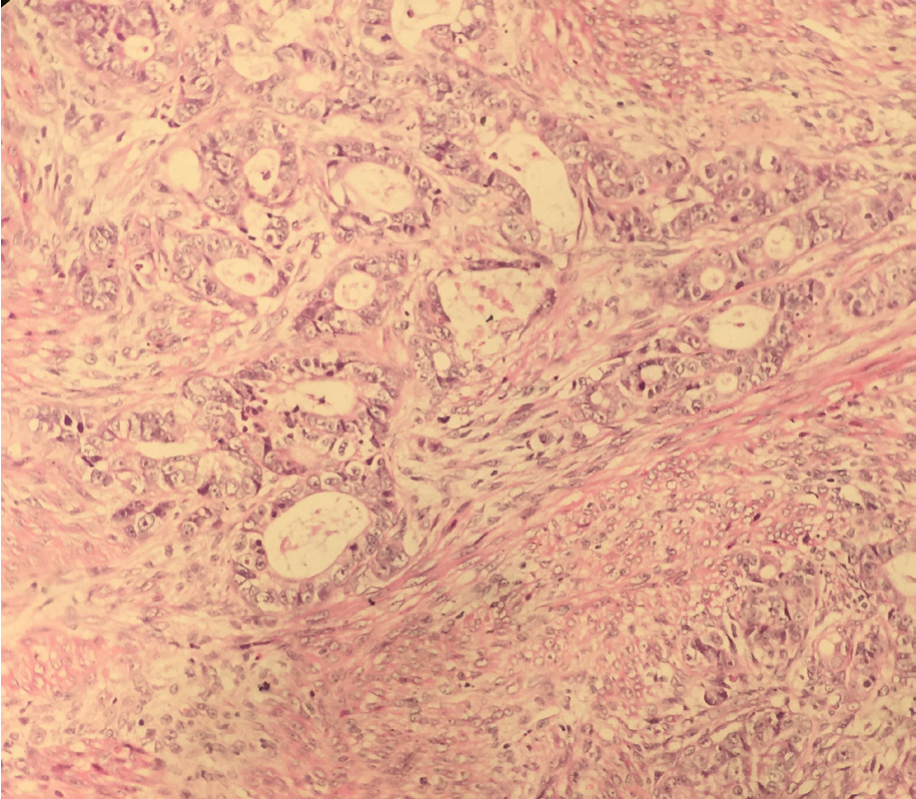
Tumor cells arranged in back-to-back glands and scattered singly (H&E, 200x).

**Figure 3.**
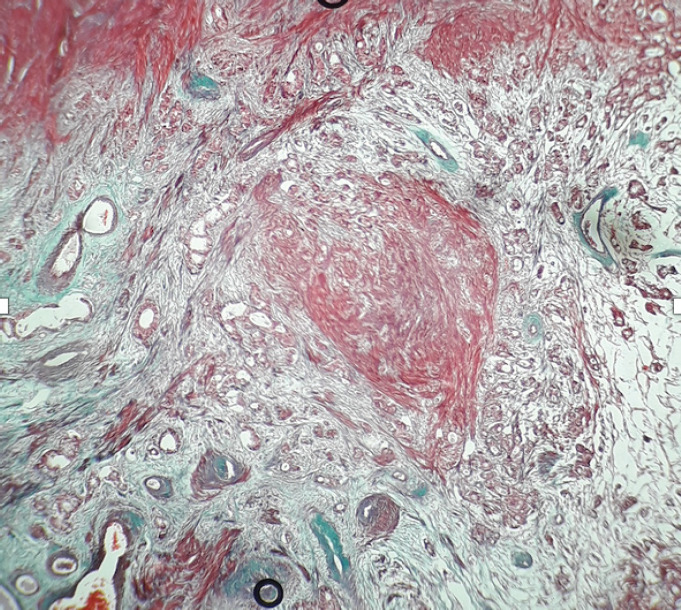
Tumor cells infiltrating the smooth muscle bundles (Masson Trichrome, 100x).

A 27-year female, a known case of Sigmoid colon, signet ring cell carcinoma diagnosed one year back for which she had undergone abdominoperineal resection. Ten months later, she presented with abdominal pain and bilateral adnexal mass, which on computed tomography (CT) scan revealed bulky bilateral ovaries with solid-cystic areas suspicious of metastatic deposits. Her laboratory investigations revealed increased serum concentration of carcinoembryonic antigen (CEA) (16.2 ng/ml) and cancer antigen (CA) 19-9 (191.69 micro/ml) with normal cancer antigen (CA) 125 (30 micro/ml). She underwent exploratory laparotomy with resection of the bilateral ovarian mass, which on histopathology revealed Metastatic adenocarcinoma on bilateral ovaries. The persistence of per vaginal bleeding, per speculum examination, revealed a large pedunculated polyp connected to the uterine fundus. On the 16^th^ postoperative day, polypectomy was done and sent for histopathology, which on microscopy revealed a feature of a leiomyoma with areas showing tumor cells infiltrating the smooth muscle bundle (Figure 1). The tumor cells are arranged in glands (Figure 2), singly scattered, some having signet ring cell morphology along with intracellular mucin. The interlacing bundles and fascicles of smooth muscle is highlighted with Masson Trichrome stain (Figure 3). Correlating with the past history and previous histopathology reports, a final diagnosis of Metastatic deposits of adenocarcinoma in submucosal leiomyoma was made.

## DISCUSSION

Colorectal carcinoma is the third most common cancer in males and second in females.^5^ It can undergo distant metastasis via hematogenous or lymphatic route. In rare cases, colorectal cancer can undergo metastasis to the female genital tract, wherein 10-80% of cases ovaries are commonly affected.^5,6^

However, metastasis to leiomyoma is uncommon. Around 19 cases of metastasis to uterine leiomyoma have been reported. The primary sites of origin being breast, colon, stomach, pancreas,^4^ whereas intracranial meningiomas are reportedly the commonest recipient neoplasm. Here we report a case of metastasis from a primary gastric cancer to a uterine lipoleiomyoma. A 65-year-old woman presented with locally advanced gastric cancer with computed tomography (CT lung, urinary bladder, thyroid^7^ and salivary gland.^8^ Metastasis from occult primary to uterine leiomyoma has also been reported.^9^ Metastasis to leiomyoma occurs either through the hematogenous route in the absence of ovarian involvement or through local lymphatic spread when ovaries are involved.^8^ As in the present case, there is metastasis to bilateral ovaries, which is a common site in female genital tracts to harbor metastasis from extragenital malignancy and submucosal leiomyoma from colon adenocarcinoma. In such a scenario it might be difficult to exclude the direct spread of tumor from ovaries to uterine leiomyoma from true metastasis from colon carcinoma. So, the differentiation of contiguous spread from tumor to tumor metastasis has been described by Pamphlett et al.^10^ that includes metastatic focus must be at least partially enclosed by a benign histologically distinct host tissue, the presence of metastasizing primary carcinoma must be proven and should be compatible with metastasis. The present case fulfills these criteria and contiguous spread from ovary has been excluded.

To our knowledge, around 104 cases of one tumor metastasizing to another tumor have been reported with various combinations of donor and recipient neoplasms.^4^ Various theories have been mentioned to describe any tumor being a common recipient tumor with special mention on meningioma as it is the commonest recipient tumor. It entirely depends on particular cancer cells (the “seeds") and tumor micro environments (the “soil"), which determine the favorable site of seeding.^11^ Some clinical and biological characteristics of the recipient tumor, such as slow growth rate, hypervascularity, high collagen, and lipid content, have also been explained to play a role in selecting a particular recipient tumor.^12^ In the present case, the metastatic deposit is present in leiomyoma, a benign tumor of smooth muscle origin and indeed a slow growing tumor, which might be a reason for leiomyoma to be one of the recipient tumors. Campbell et al. in addition has mentioned the metabolic basis for a tumor to be a common recipient tumor and have highlighted that less nutrition requirement by the recipient tumor can help flourish the survival and growth of a metastatic tumor.^13^

The use of routine imaging techniques, such as CT scan and MRI (Magnetic Resonance Imaging) can sometimes lag in excluding the presence of metastasis in an organ. So, the diagnosis relay completely on histopathological findings and proper clinical history.
